# Supplementary figures and images for: Semantic visual simultaneous localization and mapping (SLAM) using deep learning for dynamic scenes
Source: PeerJ Comput Sci. 2023 Oct 10;9:e1628. doi: 10.7717/peerj-cs.1628 (PMC10588701; doi:10.7717/peerj-cs.1628)

<https://cvg.cit.tum.de/data/datasets/rgbd-dataset/download>


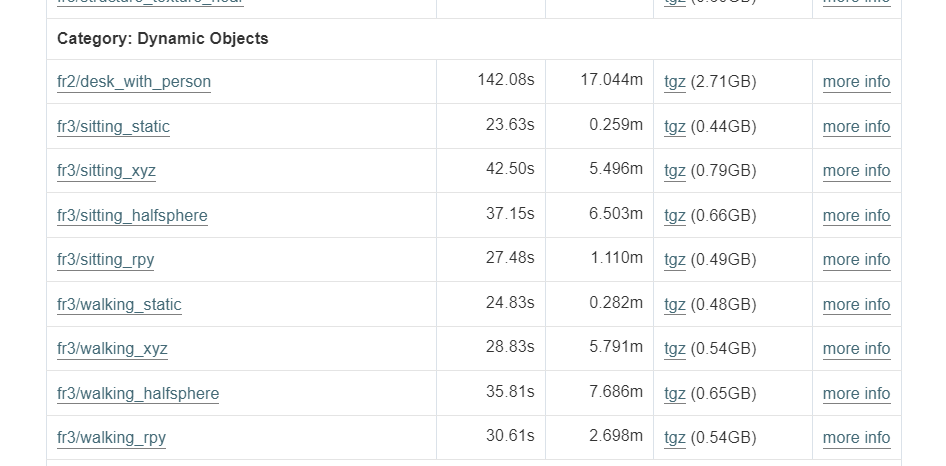


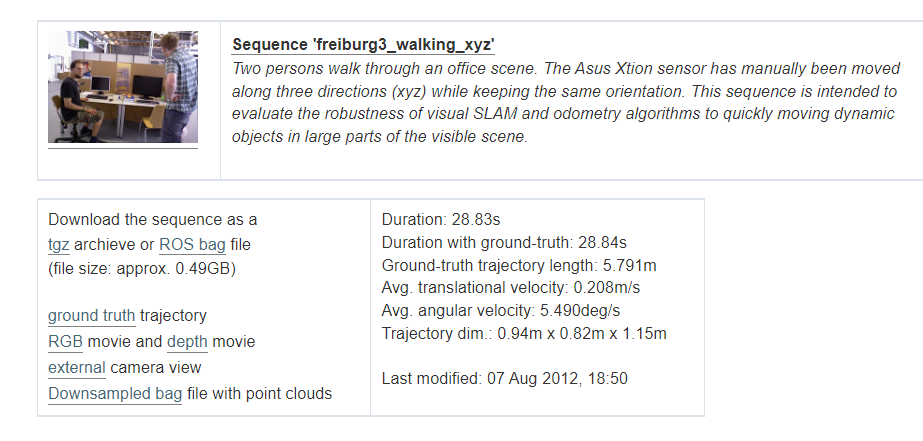

Supplement: Supplemental Information 1 [file peerj-cs-09-1628-s001.docx]
